# Purification of Islets of Langerhans from Porcine Pancreas

**DOI:** 10.1100/tsw.2002.847

**Published:** 2002-06-18

**Authors:** John Graham

**Affiliations:** School of Biomolecular Sciences, Liverpool John Moores University, UK

**Keywords:** Islets of Langerhans, transplantation, implants, diabetes OptiPrep^TM^, iodixanol, density barrier

## Abstract

Flotation through a slightly hyperosmotic discontinuous gradient iodixanol achieves a much higher recovery of islets of an improved viability than the customary method using sedimentation on to a diatrizoate/polysaccharide barrier. Flotation techniques achieve an enhanced separation of the islets from any residual digestive enzymes and from acinar cells. The method has been extended to human pancreas.

**METHOD TYPE:** extraction, isolation, purification and separation

**SUB METHOD TYPE:** centrifugation

## INTRODUCTION

Although this protocol has been developed for the isolation of islets from juvenile and adult porcine pancreas[1,2,3,4], it has been adapted to tissue from humans[5,6]. Optimal recoveries may vary with the species and tissue handling procedures and may therefore require minor adjustments to the density of the gradient solutions.

The aim of this protocol is to describe a procedure for islet purification rather than to provide a detailed account of the method of islet isolation. It is based upon an islet isolation method using the University of Wisconsin solution (UWS) as the medium for both collagenase digestion of the tissue at 37°C and for all postdigestion operations (mechanical dispersion, filtration etc) carried out at 0–4°C[1,2,3].

Some workers may prefer to restrict the use of UWS to the "cold" steps (it may be slightly cytotoxic at 37°C, or it may inhibit digestion in other species); in which case the digestion should be carried out in Hanks Balanced Salt Solutions (HBSS) or in a tissue culture medium such as RPMI (see Note 1). If such a medium is also used for the preparation of the density gradient solutions, modifications will need to be made to the volumes of OptiPrep™ and medium because these culture media have a lower density than that of UWS (see Notes 2 and 3).

The protocol uses a Working Solution containing 30% (w/v) iodixanol (osmolarity approx. 500 mOsm) produced by mixing OptiPrep™ with an equal volume of double strength UWS(2x). Gradient solutions are subsequently prepared by diluting the Working Solution with standard (1x) UWS (see Note 2). The protocol uses a 50-ml conical centrifuge tube in which the islet suspension is adjusted to $\rho$ = 1.10 g/ml (osmolarity approx. 380 mOsm) by mixing with the Working Solution and layered beneath a low-density barrier ($\rho$ = 1.09 g/ml) and a top layer of UWS. During centrifugation the islets float to the interface between the UWS and the $\rho$ = 1.09 g/ml barrier, while the acinar tissue remains in the load zone.

## MATERIALS AND EQUIPMENT

OptiPrep™ (shake gently before use)

OptiPrep™ diluent: UWS(x2): To prepare 2 l, dissolve 143.3 g of lactobionic acid (200 mM) in 1250 ml of distilled water, then adjust to pH 7.0 with 5 M KOH before adding the following in the order given (see Note 4):

13.6 g $KH_2PO_4$ (50 mM)
2.4 g $MgSO_4$ (10 mM)
71.3 g raffinose (60 mM)
0.27 g allopurinol (1 mM) (see Note 5)
3.68 g glutathione [reduced] (6 mM)
5.34 g adenosine (10 mM)
200 g pentastarch (100 g/l) (see Note 6)
Adjust with 5 M NaOH to pH 7.4 and make up to 2 l.

Diluent for gradient solutions: UWS (see Notes 6 and 7).
Filter sterilize the two diluents and store at 4°C.

Plastic conical centrifuge tubes (50 ml)
Plastic Pasteur pipette for overlayering
Low-speed (temperature-controlled) centrifuge with swinging-bucket rotor

## METHOD (summarized in Fig. 1)

1. Prepare the Working Solution ($\rho$ = 1.206 g/ml) by mixing equal volumes of OptiPrep™ and UWS(x2) and transfer 10-ml aliquots to 50-ml conical centrifuge tubes. Keep these at 4°C.
2. Prepare the low-density barrier solution ($\rho$ = 1.090 g/ml) by mixing 10-ml Working Solution with 26.36 ml of UWS and keep at 4°C (see Notes 8 and 9).
3. Digest the pancreatic tissue with collagenase in UWS (or other chosen medium) at 37°C, then carry out all subsequent operations (mechanical dispersion, filtering, etc.) in UWS at 0–4°C.

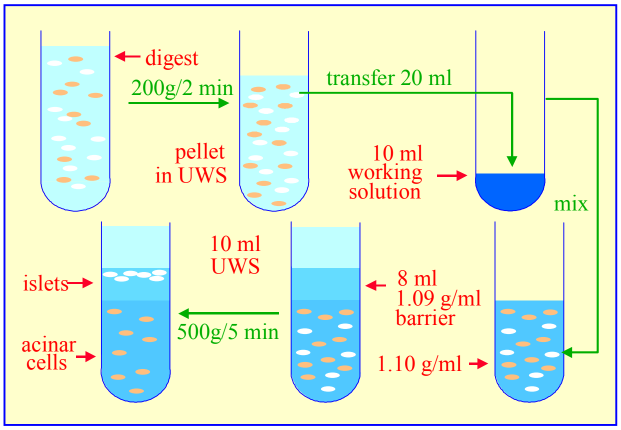

**FIGURE 1.** Islet purification flow diagram.

4. Centrifuge the digest for 2 min at 200*g* at 4°C and gently resuspend the pellet in UWS and make up to volume (a multiple of 20 ml) with this medium (e.g., 10–12 ml of packed tissue pellet in a total volume of 80 ml).
5. Transfer 20 ml of digest suspension into each of the prepared centrifuge tubes containing 10 ml of Working Solution and mix rapidly but gently by repeated inversion or pouring repeatedly between two centrifuge tubes.
6. Layer 8 ml of the low-density barrier solution over the suspension and top up the tube with 10 ml of (1x) UWS.
7. Centrifuge at 500*g* for 5 min at 4°C. The islets band at the top interface; acinar tissue remains in the load zone (see Fig. 1 and Notes 10–12).
8. Harvest the islets using a syringe and wide-bore metal cannula; dilute with an equal volume of (1x) UWS and pellet at 200*g* for 4 min.

## NOTES

1. If a medium such as HBSS or RPMI is used for the cold isolation steps, the tissue should be preincubated in cold UWS for 60 min before addition of the Working Solution. The gradient however may require significant adjustment of density and perhaps osmolality[2].
2. UWS(x2) has a density of 1.092 g/ml. Double strength HBSS or RPMI have a lower density (approx. 1.012 g/ml), consequently the amount of single-strength medium required to produce solutions of the appropriate density will require modifying (see Notes 3 and 8).
3. See Ref. [7] for more information about preparing density gradient solutions for mammalian cells.
4. Neutralization of the lactobionic acid should be carried out slowly and carefully.
5. Allopurinol is kept at the same concentration as in UWS (1x) as higher concentrations are difficult to dissolve.
6. Contact Mr. B. Henriksen (Axis-Shield PoC), Fax: +47 22 04 20 01; e-mail, bjh@no.axis-shield.com regarding commercial sources of pentastarch powder and UWS (1x).
7. UWS may be purchased commercially or it can be prepared using half the concentration of the reagents in Solution B (except allopurinol which should be at the same concentration). Alternatively it may be prepared by diluting Solution B with an equal volume of water (check pH is still 7.4), but note that the allopurinol concentration will be half that normally in UWS (1x).

8. It may be necessary to modulate the density of this layer[2] according to the isolation method that is used or if islets are purified from other species. Table 1 gives the volumes of UWS and Working Solution required to produce solutions of different densities.
9. It may be an advantage to produce the barrier solution in RPMI, this can act as a preliminary means of washing the islets free from UWS, as they float to the upper interface. Good results have been obtained with barrier solutions prepared by diluting OptiPrep™ with RPMI or RPMI containing 10% serum: 3.2 ml of OptiPrep™, and 8.8 ml of RPMI gives a solution of $\rho$ = 1.090 g/ml; if RPMI containing 10% serum is used the density is approx. 1.092 g/ml.
10. Unacceptable levels of acinar tissue contamination in the islet layer normally imply that the density of the barrier layer is too high and should be reduced.
11. The yield, purity, and viability of the islets purified by this technique show a marked improvement in all three parameters compared to those isolated by the routine Histopaque® technique (Table 2); indeed the yield of Islets from the digest is virtually doubled[1].
12. Recently it has been suggested that the purity, resistance to fragmentation, insulin response to glucose, and even the yield, are all improved by reducing the RCF to 100*g*[8]. Longer centrifugation times may consequently be required.

**TABLE 1**
**Density of Solutions Prepared from Mixing 10 ml of Working Solution (1.206 g/ml) and Different Volumes of UWS**

| UWS (ml) | Density (g/ml) |
|---|---|
| 22.65 | 1.095 |
| 31.03 | 1.085 |
| 37.06 | 1.080 |
| 45.17 | 1.075 |

**TABLE 2**
**Comparison of Islet Purification Methods**

| Purification method | Yield (% of digest) | Viability (%) | Purity (% of cells) | Size (µm) |
|---|---|---|---|---|
| OptiPrep™ | 93 | 75 | 86 | 95 (89 in digest) |
| Histopaque® | 58 | 54 | 61 | na |

na = not available.

## ACKNOWLEDGEMENTS

The author and TheScientificWorld wish to thank Axis-Shield PoC, AS, Oslo, Norway for their kind permission to adapt OptiPrep™ Application Sheet C15 in the preparation of this Protocol Article. We also thank Dr. M.P.M. van der Burg, Department of Surgery, University Hospital, Leiden, NL 2300RC, the Netherlands (e-mail: burg@surgery.azl.nl) for his help and comments in the preparation of this Protocol Article.
